# A comprehensive entomological survey and evaluation of the efficacy of different therapies on a suspected delusional parasitosis case

**DOI:** 10.1002/brb3.1945

**Published:** 2020-11-11

**Authors:** Parisa Soltan‐Alinejad, Mozaffar Vahedi, Habibollah Turki, Aboozar Soltani

**Affiliations:** ^1^ Department of Medical Entomology and Vector Control School of Health Shiraz University of Medical Sciences Shiraz Iran; ^2^ Infectious and Tropical Diseases Research Center Hormozgan Health Institute Hormozgan University of Medical Sciences Bandar Abbas Iran; ^3^ Research Center for Health Sciences Institute of Health Department of Medical Entomology and Vector Control School of Health Shiraz University of Medical Sciences Shiraz Iran

**Keywords:** delusional parasitosis, dermatology, entomological survey, Iran, psychiatric disorder

## Abstract

**Objective:**

Delusional parasitosis (DP) is one type of psychotic disorders. It is a multifactorial disorder with different etiologies. Given that very little attention is paid to entomological survey in these cases, a comprehensive study was designed and performed for the first time in Iran on a suspected DP case and its home from Shiraz during 2018–2019.

**Methods:**

In this study, entomological survey, dermatological studies, and psychological and psychiatric interventions were done respectively on a 40‐year‐old man who was referred to as a suspected case of DP.

**Results:**

No medical importance arthropods were collected from the patient's home. He was not infected with lice and other ectoparasites. Skin samples were negative for scabies, but he was infected with *Demodex folliculorum* at low level. Psychiatric studies showed that he was a secondary DP case with depression. No effect and partial remission were reported after treatment with risperidone (2–6 mg/d) and olanzapine (5 mg/d), respectively. Therapeutic effects of hypnotism were also not satisfactory.

**Conclusions:**

In Iran, the most important challenges these patients faced with are as follows: the absence of a specific referral center, patients resort to self‐treatment or traditional methods, and lack of a certain duration and dosage of antipsychotic for these cases. Comprehensive clinical trials should be done on this rare syndrome at the national level for better understanding the epidemiological profile of DP and finding the best method of treatment for Persian community.

## Graphical abstract

Entomological survey, dermatological studies, and psychological and psychiatric interventions were done on a 40‐year‐old man who was referred to as a suspected case of delusional parasitosis (DP). Psychiatric studies showed that he was a secondary DP case with depression. No effect and partial remission were reported after treatment with risperidone (2–6 mg/d) and olanzapine (5 mg/d), respectively. Therapeutic effects of hypnotism were also not satisfactory.

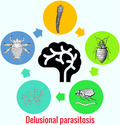

## 1 INTRODUCTION

Delusional parasitosis (DP), Ekbom's syndrome or delusions of infestation, is one type of psychotic disorders that patient believes that the small creatures are living on their skin with lack of any medical evidence (Freudenmann et al., 2007; Freudenmann, 2002a; Koo & Lee, 2001). Patients with delusional parasitosis usually refuse psychiatric referral. These patients often think they have dermatological problems, so they refer to a dermatologist (Koo & Lee, 2001).

This disorder has not had an age limit, and we can observe Ekbom's syndrome at many ranges of age (Ait‐Ameur et al., 2000). This disorder has an unknown prevalence (Boggild et al., 2010). The patients have cutaneous symptoms. They often feel biting at several parts of the body (Lee, 2008). Itching is the main symptom of these patients. Many delusional parasitosis patients believe that their neighbor's pets or birds have infested them (Hinkle, 2011; Wenning, Davy, Catalano, & Catalano, 2003). Because of scratch, some wounds appear in their skin, and in some cases, bacterial infestation is observed (Freudenmann et al., 2007).

They don't aware of their disorder, so they try many strategies for eradication and treatment of their issue such as using some dangerous pesticides or disinfectants (Lepping & Freudenmann, 2008). Sometimes, they have to change their residence several times (Lee, 2008). Because of their problem, usually people get away from them to prevent possible parasitic infestation (Lee, 2008).

Delusional parasitosis has been observed as secondary symptoms of leprosy, substance abuse, decreased visual acuity, peripheral neuropathy, and psychiatric disorders(Boggild et al., 2010; Nagaratnam & O'neile, 2000; Sugahara et al., 2000) These situations cause secondary delusional parasitosis (Boggild et al., 2010). The symptoms must be observed for at least one month (Fisher, 2008). The wounds usually present on the opposite side of the body to the patient's handedness (Freudenmann, 2002a; Lepping & Freudenmann, 2008). In these patients, to be sure dermatologists rule out any cutaneous disorder by physical examination, and then, the patient is referred to a psychiatrist (Levin & Gieler, 2013). These patients are usually resistant to psychiatric referral and psychotropic therapy.

One of the important points that are neglected is that very little attention is usually paid to entomological survey in these studies in order to identify the real causes. This comprehensive study was designed and performed for the first time in the country on a suspected case of delusional parasitosis and its home from Shiraz (capital city of Fars Province, Iran) during 2018–2019.

## 2 MATERIAL AND METHODS

### 2.1 Case description

A 40‐year‐old man with a low‐vision problem was referred to the medical entomology department of Shiraz University of Medical Sciences with a complaint of itching on his face, body, and head. He claimed there are some tiny insects in his home that they jumped and attacked his body. He was an ex‐athlete and had a good physical condition. He divorced and lived with his siblings in an almost old house.

His sister believed that at first, she did not have any similar problem but after a while because of her brother's behaviors, she felt that some parts of her body started to itch. Before referring to the Shiraz department of medical entomology, some local health experts (nonmedical entomologists) were initially examined the referred case for lice and other routine issues. After the results of the initial examination were negative, the patient was referred to the Shiraz medical entomology department for further advanced examinations.

### 2.2 Entomological survey

After referring the patient to the entomology laboratory, we thoroughly rechecked all his documentation. Data about sex, age, job, marital status, duration of symptoms, history of any psychiatric disorder, history of residual spraying in his house, all treatments that he had received, entry of secondhand items to home, and history of home reconstruction or replacement were recorded.

First of all, we checked his head for existing of any ectoparasites such as lice. After that the skin was examined to find any biting signs on his body. An expert team of entomologists was sent to the patient's home for entomological sampling. All parts in the house were checked for all macroscopic routine urban pests and vectors (Flea, louse, bedbug, hard and soft ticks, cockroaches, etc.). Furthermore, for finding any possible microscopic microorganisms (especially mites), dust samples were collected by a special vacuum cleaner.

All collected samples were transferred to the entomology laboratory and carefully were examined for finding any medical important species, including microscopic and macroscopic arthropods.

### 2.3 Dermatological studies

While doing the entomological studies, the patient was referred to a dermatologist because of having symptoms of itching on some parts of his body and face, especially on the eyelashes. After doing tests, it was observed that he had no thyroid and kidney disorders and there was no serious medical problem in his tests.

His skin was examined, and samples were taken for finding possible endoparasitic mites such as *Sarcoptes scabiei* and *Demodex spp*. and other pathogenic agents. The adhesive tape test and skin scraping were done for this purpose.

### 2.4 Psychological and psychiatric interventions

In the last phase, he referred to a psychiatrist to verify the existence of delusional parasitosis disorder in this case. Simultaneously, psychological counseling began with him by an experienced psychologist, and hypnotism was implemented as a supplementary therapeutic method in this phase of the study.

### 2.5 Ethical approval

The study was approved by the Ethics Committee of Shiraz University of Medical Sciences (Ethics code: IR.SUMS.REC. 1394. S587). Ethical considerations, including privacy of personal data, were regarded during all the steps of the research. Informed consent was obtained from all members of his family.

## 3 RESULTS

After careful inspection of the patient's home, no arthropods of medical importance were collected there except normal synanthropic species such as ants, silverfish, and spider.

Moreover, all collected samples were examined carefully by the microscope in the laboratory and no medical importance arthropods (Bloodsucking, allergenic, and parasitic species) were isolated.

After the physical examination, we could not find any evidence of arthropod biting signs on his body. In addition, he was not infected with lice and there was no blood‐feeding sign on his head. Results for scraping and for the adhesive tape tests were negative for scabies. However, he was infected with *Demodex folliculorum* at a low infestation level. After these results, he received 5% permethrin shampoo and topical crotamiton (Eurax®) cream for treatment of *Demodex* infestation. After one month of treatment, no changes were observed in the patient's itching symptoms, and patient complaints were continued about moving insects on his face and body.

After this step, the dermatologist decided to refer him to a psychiatrist for doing further studies and receiving appropriate treatments.

After reviewing the results of the patient's initial tests and interviewing him (to check the drug‐abuse history such as methamphetamine and cannabis compounds), it was determined that he had an acute delusional parasitosis syndrome with symptoms of depression. Then, psychiatrist has started second‐generation antipsychotics (SGAs), risperidone (2–6 mg/d), and olanzapine (5 mg/d) for treatment of this DP case for at least three months.

In this study, no effect and partial remission were reported after treatment with risperidone and olanzapine, respectively. Besides, hypnotism was implemented as a supplementary therapeutic method for this resistant case. Unfortunately, the curative effects of this method were also not satisfactory for this special case.

## 4 DISCUSSION

Delusional parasitosis is an uncommon but weakening syndrome characterized by the false belief of the patients that small living creatures such as parasites, mites, or vermin are infested them.

This rare syndrome occurs remarkably in middle‐aged or elderly females who live alone (R. W. Freudenmann & Lepping, 2008; Lyel, 1983; Skott, 1978; Trabert, 1995). These points are somewhat consistent with the results obtained in our study. Our patient in this study was also a middle‐aged person who did not have a partner.

It is a multifactorial disorder and also a nonspecific syndrome with different etiologies. This disease can be classified into primary and secondary DP according to the *International Classification of Diseases, 10th Revision* (Berrios, 1985; Freudenmann, 2002a; Lepping et al., 2007). After doing all phases of the research, this patient was diagnosed as a case of secondary DP because it showed a concomitant symptom of psychotic depression with delusional parasitosis.

Although it is not that difficult to diagnose, clinical management of this syndrome is profoundly problematic (Lepping & Freudenmann, 2008). In Iran, the most important challenge these patients faced with is that there is no specific referral site in the early stages of diagnosis. Patients refer to various specialists such as general practitioners, dermatologists, psychiatrists, parasitologists, medical entomologist, microbiologists, and public health staffs for diagnosis and treatment of their disease. In some cases, patients resort to self‐treatment or traditional and nonscientific methods to treat themselves. Our patient had also used some of these traditional methods that were ineffective.

These patients usually insist on "pest" treatments rather than psychiatric therapies. The more these efforts fail, the more they tend to use dangerous self‐cleansing techniques. In these conditions, often they apply several aggressive methods on their skin that causes some objective skin alterations (Freudenmann, 2002b). Our case had mild irritant contact dermatitis and scratching signs on his face and body.

Shared psychotic disorders in family member or partners happen in 5% to 18% of all cases and have been reported in many countries (Musalek & Kutzer, 1990; Trabert, 1995, 1999). In this study, after a few months, the patient's brother and sister, who lived in the same house, also developed similar symptoms but milder.

In some cases, it is really difficult to make patients participate in a research project (R. Freudenmann & Schönfeldt‐Lecuona, 2005; Munro, 1982). Fortunately, we did not encounter this problem during the study, and all the patient's family willingly assisted in all phases of the research.

In this study, no effect and partial remission were reported after treatment with risperidone and olanzapine, respectively. Besides, hypnotism was implemented as a supplementary therapeutic method for this resistant case. Unfortunately, the therapeutic effects of this method were also not satisfactory for this special case.

Another challenging point in the clinical management of DP is finding the best duration and dosage of antipsychotic treatment (Hamann & Avnstorp, 1982). In the present study, two available medications with a variety range of duration and dosage were used, but no effect and partial remission were reported after treatment with risperidone and olanzapine, respectively.

## 5 CONCLUSION

In this resistant case, full remission with antipsychotic treatments and other implemented methods failed. This could be attributed to some reasons that the most important is the absence of relevant clinical trials on the use of available medications, especially second‐generation antipsychotics in DP on the target population of Iranian patients.

In this regard, it is suggested that comprehensive clinical trials should be done on this rare syndrome at the national level for better understanding the epidemiological profile of DP and finding the best method of treatment for Persian community.

## CONFLICT OF INTEREST

No conflict of interest to declare.

## AUTHOR CONTRIBUTIONS

A. S. and P. SA conceived and designed the study. M. V., H. T., and P. SA gathered data. A. S. interpreted data and critically revised the manuscript for important intellectual content. P. SA., M. V., H. T., and A. S drafted the manuscript.

### Peer Review

The peer review history for this article is available at https://publons.com/publon/10.1002/brb3.1945.

## Data Availability

The data that support the findings of this study are available on request from the corresponding author. The data are not publicly available due to privacy or ethical restrictions.
